# Influence of microplastics on small-scale soil surface roughness and implications for wind transport of microplastic particles

**DOI:** 10.1098/rsta.2024.0446

**Published:** 2025-10-23

**Authors:** Annie Ockelford, Joanna Bullard, Cheryl McKenna Neuman, Patrick O'Brien

**Affiliations:** ^1^ University of Liverpool Faculty of Engineering, Liverpool, England, UK; ^2^ Department of Geography and Environment, Loughborough University, Loughborough, Leicestershire, UK; ^3^ Trent Environmental Wind Tunnel, Trent University, Peterborough, Ontario, Canada

**Keywords:** wind erosion, soil surface roughness, microplastic entrainment, microplastic shape, microbead, fibre

## Abstract

Microplastics are an anthropogenic contaminant widely recognized for their effect on marine and freshwater systems, but their terrestrial effects remain less well studied. The inclusion of microplastics in soils has the potential to affect a range of different soil properties, including bulk density, hydraulic conductivity and aggregation. Soil properties affect the susceptibility of soils to wind erosion, and it is therefore likely that where the quantity of microplastics present in soils is sufficient to change soil properties, it may also change the response of soils to wind erosion. This paper quantifies whether the presence of microplastics in sediments affects the development of small-scale soil surface roughness (SSR) properties during wind erosion, and whether there are any relationships between indices of SSR and microplastic flux due to wind erosion. Two contrasting substrates (well-sorted sand and poorly sorted soil) and two types of microplastic (polyethylene beads and polyester fibres) are used. SSR is quantified using geostatistically derived indicators calculated from high-resolution laser scans of the soil surface with and without microplastics, and before and after wind erosion simulated using a wind tunnel. Our results reveal the relative size of the microplastic to the mineral sediment is key to controlling microplastic flux.

This article is part of the Theo Murphy meeting issue ‘Sedimentology of plastics: state of the art and future directions’.

## 1. Introduction

Microplastics are solid, synthetic-polymer-containing particles less than 5 mm in size (NOAA/European Marine Strategy Directive [1]), which are increasingly recognized as an anthropogenic contaminant that can have substantial environmental effects [2]. Extensive research has been undertaken on the identification and detection of microplastics in marine and freshwater aquatic environments [3,4], but while the presence of microplastics in soil and terrestrial ecosystems is recognized, there has been considerably less research regarding their effects [5–8].

Microplastics derive from land-based anthropogenic activity, with more than 50% of all microplastics estimated to be retained in the terrestrial environment for long periods of time [9,10]. Microplastics are incorporated in managed soils via the application of compost [11], sewage sludge [12,13] and incomplete removal, or breakdown, of agricultural plastic mulch films [14,15]. They can also be deposited on soils through flooding [16,17] or via atmospheric deposition [13,18]. Concentrations of microplastics in soils vary depending on geographical context and location [19] but can reach more than 200 000 particles kg^−1^ [15,20] and over 6% by weight in heavily contaminated areas [21].

The inclusion of microplastics in soils has the potential to affect a range of different soil properties, including bulk density, water holding capacity, hydraulic conductivity, aggregation and microbial activity [22]. The introduction of microplastics, which are comparatively less dense than most natural soil minerals, can cause a decrease in the bulk density of soils [22,23] and may also affect the size of pore spaces and the interaction of mineral particles within the soil [13]. Several studies suggest the size profile of soil aggregates is altered when microplastics are introduced to soils. This is more often reported as a reduction in the proportion of large aggregates [22,24,25], but in some case studies, particle aggregation was observed to increase [26], and effects may depend on experimental design, organic matter content and the presence or absence of bioturbating organisms [13,27]. The effect of microplastics in soils varies dependent on the concentration of plastic and the relative sizes and shapes of the microplastic and mineral particles. For example, microplastic fibres are thought to have higher potential to change soil properties, such as soil structure and bulk density, than beads because the flexible and linear structures of fibres are substantially different to those of typical soil mineral particles [6,22]. Partially embedded elongated particles may protrude into the airflow and may also be flexible and can wrap around mineral particles contributing to the aggregation of soil particles [22].

Soil properties affect the susceptibility of soils to wind erosion, primarily via effects of surface roughness [28–30]. Accounting for surface roughness effects on aeolian transport is a key component of the model uncertainty [31–33]. Specifically, the spatial distribution of roughness elements has a significant effect on aeolian sediment flux by influencing the magnitude and distribution of shear stress over a surface [34]. Römkens & Wang [35] identified different scales of surface roughness, the smallest two of which are determined by the size of the primary soil particles (less than or equal to mm scale) and the size of soil aggregates (mm); soils containing smaller particles, such as the sand beds used in these experiments, are more susceptible to wind erosion than those with a greater proportion of, or larger, aggregates as in the soil beds used for this study [36,37]. The third scale described by Römkens & Wang [35] relates to the organization of grains to form microtopography (cm).

It is therefore likely that where the quantity of microplastics present in soils is sufficient to change soil properties via a change to surface roughness, it may also change the response of soils to wind erosion. There has been very little research concerning the wind erosion of soils containing microplastics, but both Rezaei *et al*. [37] and Bullard *et al*. [38] found that, for a mixed soil–microplastic bed and a controlled wind regime, microplastics were entrained preferentially compared to mineral soil particles. The incorporation of microplastics into soils and their subsequent removal may be expected to alter the small-scale surface roughness and structural properties of the soils, and this is likely to vary with both microplastic type and the nature of the substrate [39]. For example, the most common microplastic shapes found in soils are fibres, fragments and films [40,41], and these have the potential to affect soil surface roughness (SSR) because the plastics are non-spherical and deviate from typical mineral particle shapes.

In this paper, we report the results from a series of wind-tunnel experiments designed to determine, first, whether the presence of microplastics in sediments affects the development of small-scale SSR properties during wind erosion and, second, whether there are any relationships between indices of SSR and microplastic flux as a result of wind erosion. We use non-contact laser scanning to capture changes to soil microtopography (less than 10 cm^2^) because SSR, even at the smallest scales, plays a role in modifying exchanges between terrestrial and atmospheric systems [42,43] and can influence trapping and ejection of particles. Our findings indicate the extent to which the presence and influence of microplastics on small-scale SSR evolution need to be accounted for in models of aeolian particle transport.

## 2. Material and methods

### (a) Material

Experiments were performed in the Trent University Environmental Wind Tunnel (TEWT), a boundary-layer simulation tunnel with an open-loop suction design. The tunnel working section is 13.8 m long, with a cross section 0.7 m wide by 0.76 m high, and the whole tunnel is housed in an environmental chamber, which, for this study, was held constant at 20°C and 20% relative humidity to minimize electrostatic effects. Further details of the TEWT facility are provided in previous publications [44–46].

**Figure 1.**
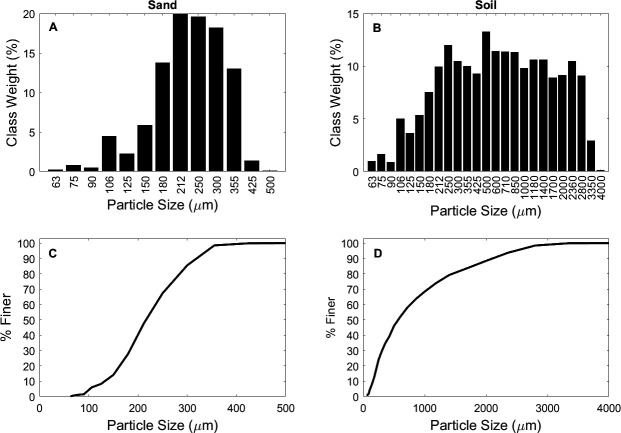
(A) Grain size distribution by class weight (A,B) and cumulative distribution (C,D) of substrate one (sand) and substrate 2 (soil)*.*

Experiments were conducted using two substrates and two types of microplastics. Substrate one was well-sorted quartz sand (hereafter ‘sand’, particle density of 2.65 g cm^3^ and median grain size (*D*_50s_) of 215 μm) similar to that found in sandy agricultural and rangeland soils [37] as well as on beaches [47] and sandy lakeshores [48] from which high concentrations of macroplastics and microplastics have been reported (figure 1A). Substrate two was a poorly sorted soil (hereafter ‘soil’ with median grain size (*D*_50s_) of 500 μm) containing 13.2% organic matter (calculated by loss on ignition at 850°C; figure 1B), more typical of agricultural soils [49].

Two types of microplastics were used in the experiments. The first of these was polyethylene microspheres in the size range 212−250 μm with a density of 1.2 g cm^3^ (hereafter ‘beads’). The beads were primary microplastics and, based on the literature, hypothesized to have a minimal effect on SSR due to their similarities in shape to mineral soil particles [23]. The second were polyester fibres (hereafter ‘fibres’) approximately 5 mm long with a width of 0.5−1 mm and a density of 1.38 g cm^3^. The fibres were secondary microplastics that are more commonly found in the field and in association with airborne deposition and transport [41,50–52]. Plastics were mixed with the substrate at concentrations of 40, 240, 640 and 1040 mg kg^−1^_dw_, which are representative of the range of microplastic concentrations reported from terrestrial soil samples [19].

### (b) Methods

**Figure 2.**
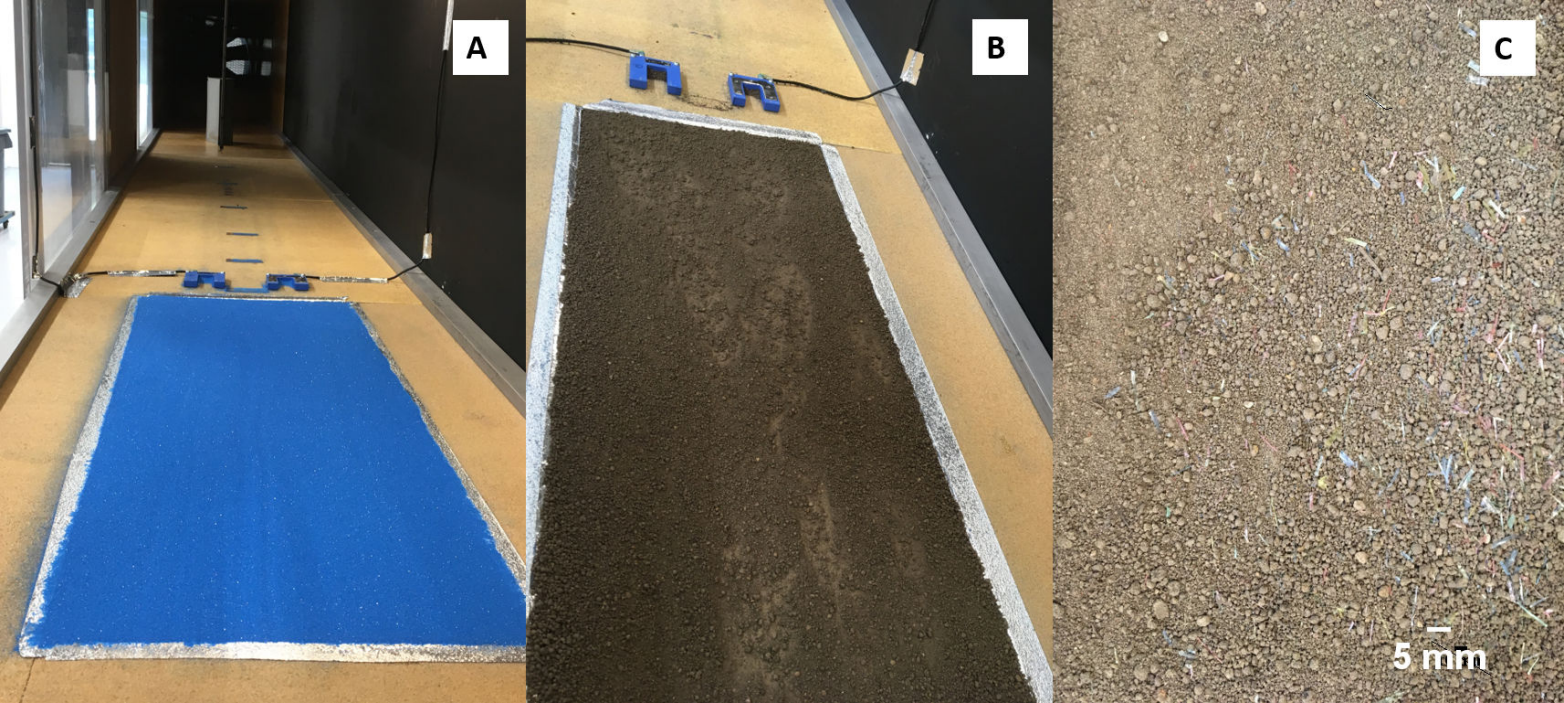
(A,B) Looking downwind at the wind-tunnel set-up for the sand (A) and soil (B) beds including the pair of Wenglor sensors. The sediment trap at the downwind end of the tunnel and the glycerol-coated microscope slides attached to the bed surface downwind of the tray are also shown in (A). (C) Example of the soil bed seeded with the microplastic fibres*.*

For each experiment, test surfaces were prepared by filling a 1.0 × 0.35 × 0.025 m metal tray with the given mixture of test material (substrate, or substrate + microplastic), which was prepared by weighing and manually mixing the appropriate ratios of substrate to plastic. The test material was levelled such that the substrate surface was flush with the tunnel floor and finally an airtight seal was secured around the tray perimeter (figure 2). The upwind fetch was 8 m, and airflow travelled over a loose bed of randomly distributed gravel to adjust itself and achieve the desired aerodynamic roughness. For each experiment, free-stream wind speed was increased incrementally by 0.25 m s^−1^ every 30 s from well below threshold until it reached 14 m s^−1^, and the whole bed was experiencing continuous saltation at which point the experiment was stopped. The onset of particle transport was recorded using two fork-type Wenglor sensors downwind of the test bed. Typically, active particle transport only lasted 4−8 min during each experiment, and the Wenglor’s remained exposed and actively measuring throughout. Each experiment was repeated three times.

#### (i) Soil surface characterization

Soil surfaces were characterized using a high-resolution 3D Konica Minolta Vivid9i laser scanner. The scanner was mounted above the test bed with a viewing area of approximately 150 mm by 108 mm at a horizonal and vertical resolution of 0.25 and 0.5 mm, respectively. The sediment surface was scanned before and after each of the experiments to enable changes in soil microtopography due to wind erosion to be determined. The tray edges were included in each scan to ensure that repeat scans could be aligned. Once aligned, a 100 × 100 mm area in the centre of each scan was extracted for analysis to minimize edge effects.

The laser scan data were post-processed by linear interpolation onto a 0.25 mm grid using MATLAB to produce digital elevation models (DEMs) of each soil surface. On a few occasions, the laser beam was occluded, returning no data for a given point. These null data points were returned as ‘NaN’ values and were replaced by an average value calculated from the surrounding eight cells (1.2% of all data points). All the elevation (*z*) data were detrended using a linear model to remove any large-scale oriented slope issues potentially introduced during the screeding process that may create bias in the metrics derived from them.

#### (ii) Indices of soil roughness and spatial variability

SSR was characterized using both aspatial and spatial indices, which have been demonstrated to be effective for quantifying small-scale physical changes to soil surfaces in response to geomorphic processes [53–57]. Aspatial metrics quantify bulk properties of the surface topography and include topographic range (TR) and random roughness (RR). The TR of each soil scan indicates the difference in height (mm) between the lowest and highest detected points in the DEM. The RR is the s.d. in height after eliminating oriented roughness such as slope [58,59].

To capture spatial complexity, techniques such as semi-variogram analysis are widely used [53,60]. Semi-variograms provide a measure of the correlation of the surface elevations in different directions at different spatial lags. In this study, for each scan, a one-dimensional semivariogram was produced to which a spherical model was fitted. This was chosen for its known suitability for describing datasets with high short-range variability, linear behaviour close to the origin and where an asymptote (sill) is reached at small ranges; full details can be found in Corwin *et al*. [61] and Bullard *et al*. [55]. Model results were used to determine geostatistically derived indicators. The spatial range (*a*) indicates the maximum scale of spatial variation in the data such that larger values of *a* are associated with larger scales of spatial patterning. Total variance (sill) describes the total amount of spatial variation in the data such that, for the same scale, more spatially varied surfaces have higher values.

#### (iii) Quantification of particle flux

To relate changes in SSR to both sediment and microplastic removal, downwind particle flux was measured using glycerol-coated microscope slides. Slides were attached to the bed surface downwind of the tray at distances of 5, 30, 60, 100, 150 and 200 cm and were used to trap transported particles. Using image analysis, the total area of sediment and number and area of microplastics captured on the glycerol slides were calculated, which allowed microplastic enrichment ratios to be calculated. The enrichment ratio was defined as the ratio of microplastic content in the particulate matter transported during threshold tests as compared to that in the original substrate [38]. If the quantities of entrained sediment and microplastics were small, the material captured on the glycerol-coated slides were bulked together for replicate tests.

## 3. Results

### (a) Aspatial soil surface roughness characteristics

**Figure 3.**
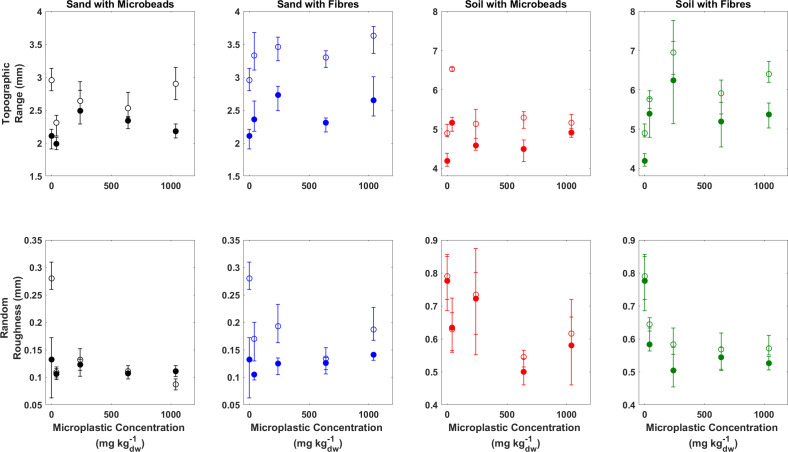
Summary of the relationship between TR (top row) and RR (bottom row) prior to (closed symbols) and after (open symbols) wind erosion. Error bars in each represent the maximum and minimum values derived from the three repeat experiments.

Prior to wind erosion, the TR (the difference between the highest and lowest elevations), a surrogate for surface roughness, was 2.54 mm for sand beds and 6.67 mm for soils beds where there was no plastic. For both substrates and all treatments, topographic roughness increased following wind erosion (figure 3 and table 1). The increase in TR was 0.5 mm for sand beds and 0.19 mm for soil beds containing no plastic. Microplastic type appears to have an effect on results, but there is no systematic relationship between change in TR and microplastic concentration (figure 3 and table 1), and hence the change in TR before and after wind erosion has been averaged for all concentrations. Specifically, in sand beds, TR increases on average by 0.54 (*σ* = 0.61) and 0.92 mm (*σ* = 0.13) for beds containing beads and fibres, respectively. In soil beds, TR increases on average by 0.71 (*σ* = 0.27) and 0.74 mm (*σ* = 0.48) for beds containing beads and fibres, respectively.

**Table 1.** Aspatial topographic indicators before and after wind erosion. The ${∆}_{Tr}$ and ${∆}_{RR}$ values have been, as the difference between the TR and RR before wind erosion, subtracted from the TR and RR after wind erosion. The $\Delta_{TR}/D_{50s}$ and *$\Delta_{TR}/D_{50p}$*is the ratio of the change in TR (Δ_TR_) to the median sediment size (*D*_50s_) and the median plastic size (*D*_50p_).

		av. TR (mm)										av. RR (mm)					
substrate	concentration (mg kg^−1^_dw_)	bead					fibre					bead			fibre		
		before	after	*Δ_TR_*	Δ_*TR*_*/D*_*50s*_	Δ_*TR*_*/D*_*50p*_	before	after	Δ_*TR*_	Δ_*TR*_*/D*_*50s*_	Δ_*TR*_*/D*_*50p*_	before	after	Δ_*RR*_	before	after	Δ_*RR*_
sand	0	2.54	3.04	0.5	2.33	n/a	2.54	3.04	0.5	2.33	n/a	0.13	0.23	0.1	0.13	0.23	0.1
	40	1.99	2.31	0.32	1.49	1.39	2.36	3.33	0.97	4.51	1.29	0.11	0.21	0.1	0.1	0.17	0.07
	240	2.45	2.64	0.19	0.88	0.83	2.73	3.46	0.73	3.4	0.97	0.12	0.2	0.08	0.13	0.19	0.06
	640	2.34	2.53	0.19	0.88	0.83	2.31	3.3	0.99	4.6	1.32	0.11	0.16	0.05	0.13	0.13	0
	1040	2.18	3.63	1.45	6.74	6.3	2.65	3.63	0.98	4.56	1.31	0.11	0.12	0.01	0.14	0.19	0.05
soil	0	6.67	6.86	0.19	0.38	n/a	6.67	6.86	0.19	0.38	n/a	0.78	0.83	0.05	0.78	0.79	0.01
	40	5.39	5.76	0.37	0.74	1.61	5.16	6.53	1.37	2.74	1.83	0.63	0.71	0.08	0.58	0.64	0.06
	240	6.24	6.95	0.71	1.42	3.09	4.58	5.13	0.55	1.1	0.73	0.72	0.78	0.06	0.5	0.58	0.08
	640	5.19	5.9	0.71	1.42	3.09	4.49	5.29	0.8	1.6	1.07	0.5	0.54	0.04	0.54	0.57	0.03

Research has previously suggested that relative size effects between the median microplastic and median soil particle diameter (*D*_50p_/*D*_50s_) are important controls on microplastic entrainment by wind [62,63], but the effect of topographic development has not been quantified. This is despite the recognition that relative roughness in soils has been shown to be important in determining soil structure interactions and erosion (e.g. [64]). In experiments reported here, the initial TR is very different for the two substrates, and it is therefore useful to analyse relative roughness changes in response to wind erosion and microplastic type relative to both the median bed sediment size ($\Delta_{TR}/D_{50s}$) and the median microplastic size ($\Delta_{TR}/D_{50p}$). This parameter is widely used in the fluvial geomorphology literature (e.g. [65]) but not yet in the soil’s literature despite obvious comparisons. As discussed above, given there was no obvious effect of microplastic concentration, data were averaged for all concentrations (table 1).

In sand beds, $\Delta_{TR}/D_{50s}$ values are 2.33 where no microplastics are present as compared to an average relative roughness of 2.50 and 4.27 for sediment beds containing beads and fibres, respectively. In sand beds, the $\Delta_{TR}/D_{50s}$ are 1.71 times greater for experiments using fibres compared to those using beads, suggesting that fibres contribute towards the development of roughness elements at the surface. In comparison, for soil beds, the $\Delta_{TR}/D_{50s}$ values are 0.38 where no microplastics are present as compared to 1.41 and 1.49 for beds containing beads and fibres, respectively. When considering $\Delta_{TR}/D_{50p}$, the average relative roughness is 2.34 and 1.22 in sand beds containing beads and fibres, respectively, as compared to 3.07 and 0.99 for soil beds containing beads and fibres, respectively. This suggests that the scale of the roughness developed is larger than the beads but similar in scale to that of the fibres.

RR is an indicator of topographic variability and is up to 7.1 times higher for the soil than for the sand beds before wind erosion (figure 3 and table 1). As with TR, wind erosion causes an increase in RR for all experiments, but there is no clear influence of microplastic concentration and nor does there appear to be an effect caused by microplastic type; average change in RR is 0.06 mm for all beds irrespective of sediment type or microplastic type.

**Figure 4.**
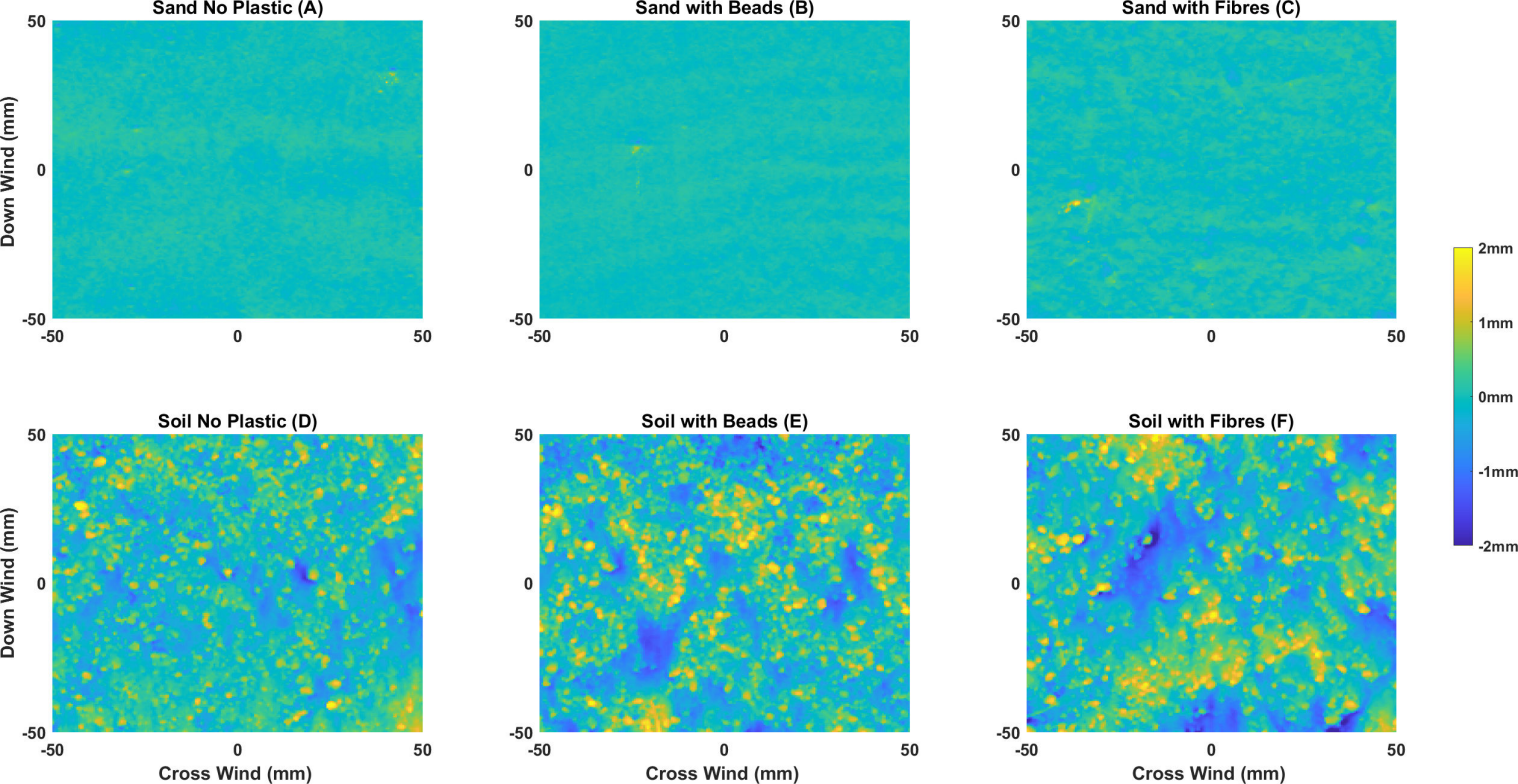
DEMs of the surfaces at the end of the experiments for microplastic concentrations of 0 (A,D) and 1040 mg kg^−1^_dw_ (B–C and E–F). The beds produced in this run are similar in terms of their spatial patterning to those observed to develop in all other replicate runs. Wind direction is from top to bottom of the images.

Given that the larger relative changes in topographic roughness for the sand beds cannot be attributed to the type or concentration of plastics contained within the bed, they are likely to be associated with the initiation of transverse sand ripples [66]. These can be seen in figure 4, which shows the post-wind erosion DEMs of the sediment beds. Proto-ripples are visible as ridges oriented perpendicular to airflow, which will increase the large-scale topographic roughness. By comparison, the surfaces of the soil beds, irrespective of the concentration or type of microplastic, are more topographically complex compared to the sand beds but do not exhibit oriented roughness features. This complexity probably reflects the wider range of grain sizes in the soil (figure 1) and the presence of soil aggregates at the surface, visible on the bed surface (figure 4D–F). A one-way analysis of variance using surface elevations reveal there is no statistical difference caused by differing concentrations of microplastics for either sand or soil beds prior to wind erosion. Furthermore, there is no statistical difference between surfaces before and after wind erosion for either type of microplastic, except for sand beds that contain fibres (*p* values range between 0.041 and 0.049).

### (b) Spatial surface roughness characteristics

While the aspatial metrics indicate differences in the topographic development of sand and soil beds in response to wind erosion, they give no indication of the spatial complexity or structure of that topography. However, visual analysis of the DEMs (figure 3) suggests there are differences in both the scale and type of surface roughness elements that develop in response to wind erosion. The spatially derived indicators of the sill (the total amount of spatial variability) and the spatial range (the extent of spatial correlation) can be used quantify the degree and scale of the spatial complexity.

**Figure 5.**
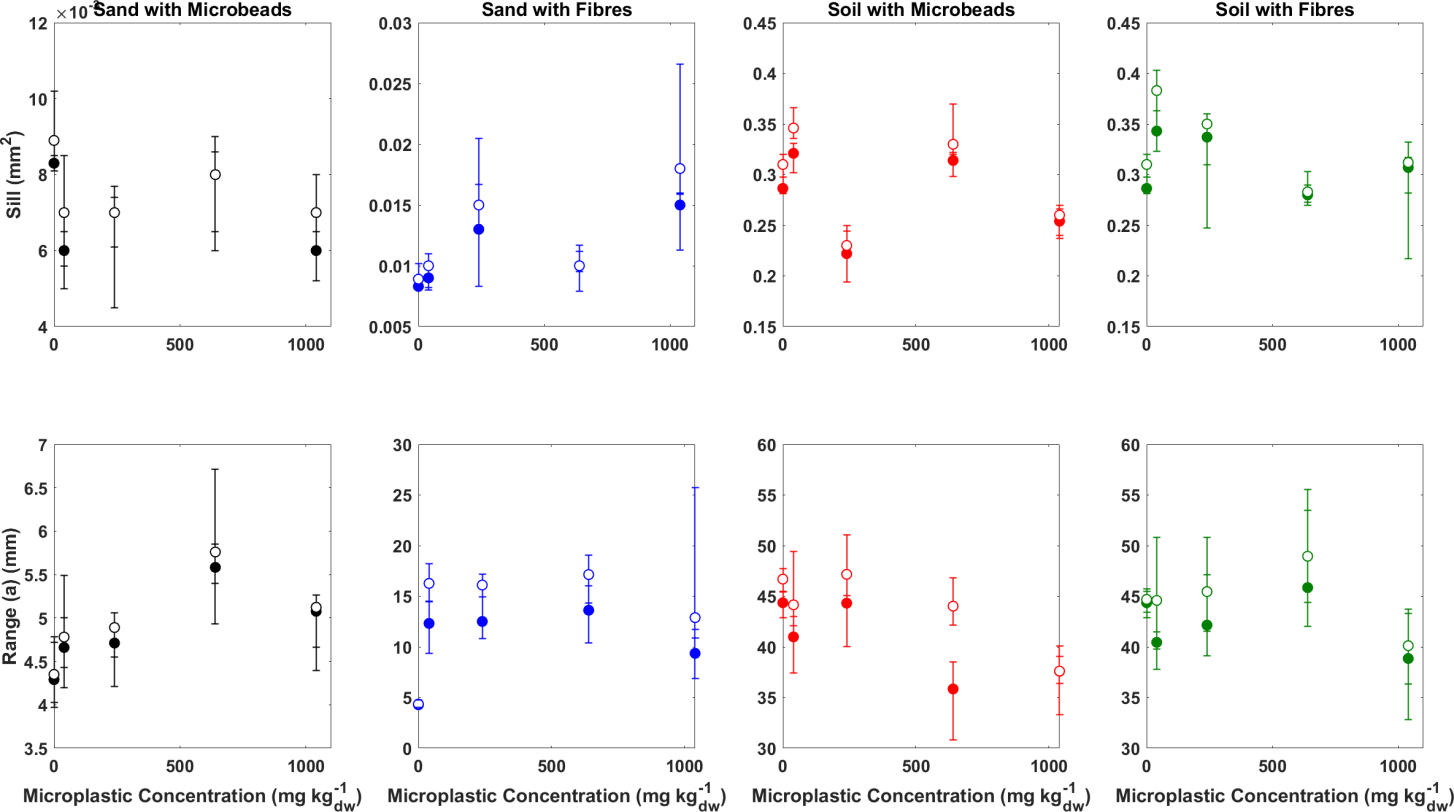
Summary of the relationship between sill (top row) and range (bottom row) prior to (closed symbols) and after (open symbols) wind erosion. Error bars in each represent the maximum and minimum values derived from the three repeat experiments.

Prior to erosion by wind of sand beds containing no plastic, the sill is 0.008 mm^2^ and an average of 0.007 (*σ* = 0.001) and 0.012 mm^2^ (*σ* = 0.002) for sand beds containing beads and fibres, respectively. After wind erosion, the sill does increase, suggesting an increase in the degree of spatial variability and sediment beds containing fibres undergo a greater change than beds containing beads (0.001 versus 0.002 mm^2^), although again the concentration of the plastics within the bed does not appear influential. In soil beds, the spatial variability is an order of magnitude higher than in sand beds (figure 5 and table 2): 0.29 mm^2^ in soil beds containing no plastics and an average of 0.28 (*σ* = 0.01) and 0.32 mm^2^ (*σ* = 0.02) for soil beds containing beads and fibres, respectively. After wind erosion, soil beds exhibit the same pattern as sand beds where spatial variability increases, but beds containing fibres exhibit greater changes than beds containing beads (table 2).

**Table 2.** Spatial topographic indicators before and after wind erosion. The Δ_s_ and Δ_a_ values have been, as the difference between the sill and range before wind erosion, subtracted from the sill and range after wind erosion.

		av. sill (mm)						av. range (a) (mm)									
substrate	concentration (mg kg^−1^_dw_)	bead			fibre			bead				fibre					
		before	after	Δ_s_	before	after	Δ_s_	before	after	Δ_a_		before		after		Δ_a_	
sand	0	0.008	0.009	0.001	0.008	0.009	0.001	4.29	4.35	0.06		4.29		4.35		0.06	
	40	0.006	0.007	0.001	0.009	0.010	0.001	4.66	4.78	0.12		13.32		16.27		2.95	
	240	0.007	0.007	0.000	0.013	0.015	0.002	4.71	4.89	0.18		12.51		16.11		3.60	
	640	0.008	0.008	0.000	0.010	0.010	0.000	5.58	5.76	0.18		13.62		17.14		3.52	
	1040	0.006	0.007	0.001	0.015	0.018	0.003	5.08	5.12	0.04		9.36		12.89		3.53	
soil	0	0.286	0.310	0.024	0.286	0.310	0.024	44.36	46.69	2.33		44.36		46.69		2.33	
	40	0.321	0.346	0.025	0.343	0.383	0.040	40.99	44.15	3.16		40.45		44.59		4.14	
	240	0.222	0.230	0.008	0.337	0.350	0.013	44.32	47.17	2.85		42.15		45.56		3.41	
	640	0.314	0.330	0.016	0.280	0.283	0.003	35.85	44.02	8.17		45.58		48.95		3.37	
	1040	0.254	0.260	0.006	0.307	0.312	0.005	37.59	37.61	0.02		38.58		40.12		1.54	

Prior to wind erosion, the extent of spatial correlation, described by the range (*a*), is 4.29 mm for sand beds containing no microplastics and an average of 5.01 (*σ* = 0.07) and 12.20 mm (*σ* = 0.30) for beds containing beads and fibres, respectively (figure 5 and table 2). After wind erosion, the extent of the spatial correlation increases by 0.06 mm for sand beds containing no plastics but doubles for beds containing beads (av, 0.13 mm, *σ* = 0.07) and is 56 times higher for sand beds containing fibres (av, 3.4 mm, *σ* = 0.30). The range is an order of magnitude higher in the soil beds with values of 44.36 mm for beds containing no microplastics and an average range of 39.69 (*σ* = 3.75) and 41.69 mm (*σ* = 2.98) for beds containing beads and fibres, respectively. After wind erosion, the extent of the spatial correlation increases by 2.33 mm for beds containing no plastics and is 1.5 time higher for beds containing beads (av, 3.55 mm, *σ* = 3.39) and 1.34 times higher for beds containing fibres (av, 3.12 mm, *σ* = 1.11). These data suggest that wind erosion of sand beds show the greatest changes to the extent of spatial correlation, which is particularly observed when fibres are present. Conversely, in soil beds, the addition of microplastics makes very little difference to the extent of spatial correlation.

### (c) Relationships between soil surface roughness indices and microplastic flux

Both the aspatial and spatial characterizations of the sediment bed surfaces suggest that surface microtopography is controlled primarily by mineral grain size, but that plastic type may exert an influence, particularly within sand beds.

**Figure 6.**
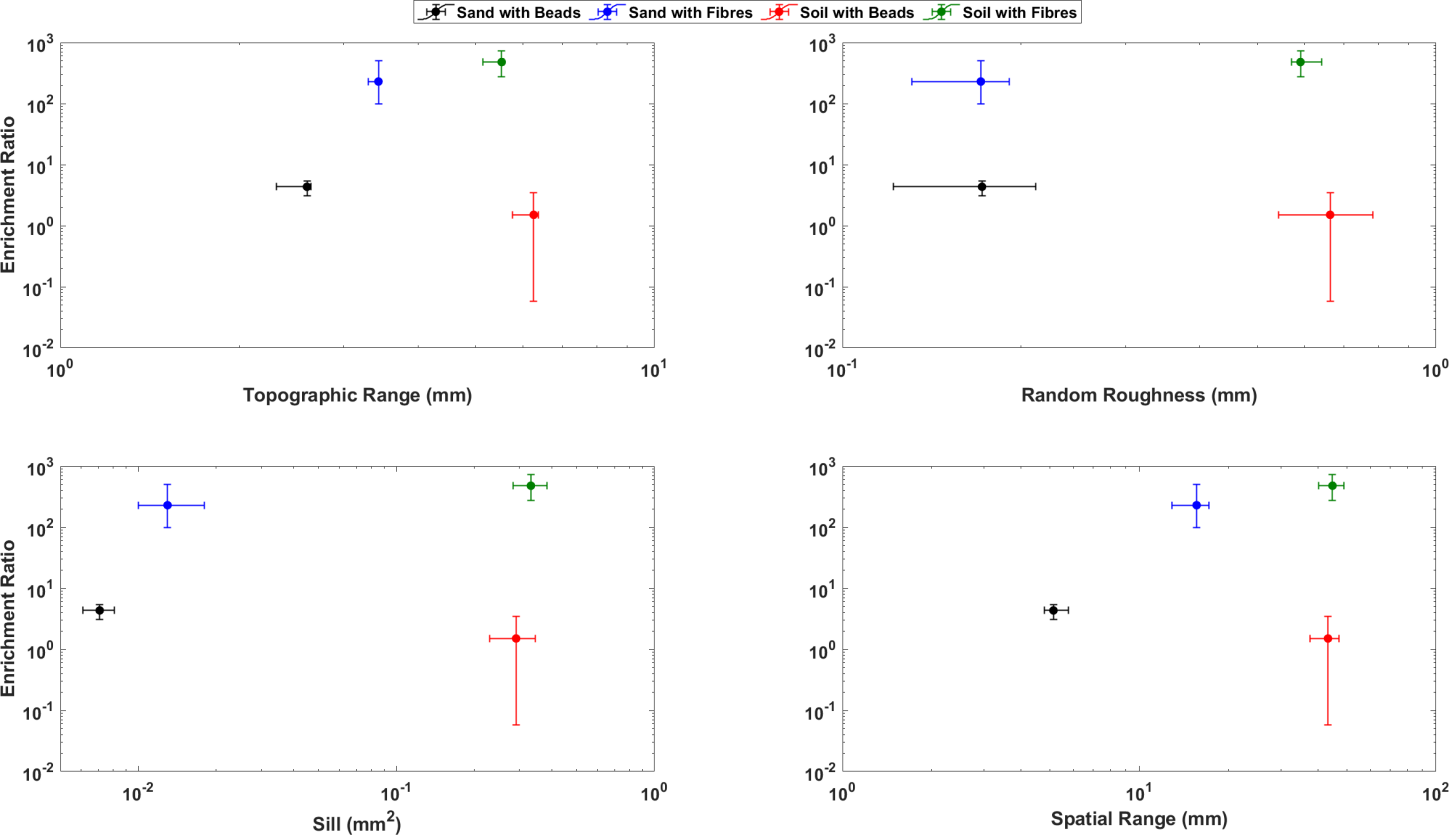
Summary of the relationships between enrichment ratios and aspatial (top row) and spatial (bottom row) descriptors of SSR. Data points depict the concentration averaged parameter with error bars in each representing the maximum and minimum values derived from the three repeat experiments. All data for the four descriptors of SSR are calculated from post-wind erosion surfaces, and the enrichment ratio is defined as the ratio of microplastic content in the particulate matter transported during threshold tests as compared to that in the original substrate [38].

To explore the relationship between surface evolution and microplastic flux, the concentration averaged aspatial and spatially correlated variables have been plotted as a function of the concentration averaged enrichment ratio of microplastics in the windblown sediment (figure 6). As described in Bullard *et al*. [38], mineral particles and microplastics were transported during all the experiments, but the material transported was enriched in plastics compared to the original substrate concentration, and enrichment was an order of magnitude higher for the fibres for both bed substrates compared to beads (figure 6). Enrichment ratios in sediment beds containing beads are smaller where surfaces are rougher and more spatially complex. Conversely, enrichment ratios for sediment beds containing fibres are higher where soils surface are rougher and more spatially complex.

## 4. Discussion

### (a) Surface development in response to wind erosion

The susceptibility of soils to wind erosion is strongly controlled by surface roughness [28–30]. At the smallest scales, surface roughness is determined by primary soil particle size (less than or equal to mm scale) and the size of soil aggregates (mm); soils containing smaller particles, such as the sand beds used in these experiments, are more susceptible to wind erosion than those with a greater proportion of, or larger, aggregates as in the soil beds used for this study [36,37].

As roughness scales increase, the organization of grains to form microtopography (cm) becomes important [35]. In the experiments reported here, both sand and soil surfaces become rougher when subjected to wind erosion, which has been demonstrated in other studies [40,67,68]. The larger grains and aggregated particles present in the soil bed created a higher initial surface roughness than in the sand bed, but the relative increase in roughness in response to wind erosion was greater in the sand bed than the soil. This greater change has been attributed to the development of sand ripples [69].

In addition to absolute changes to surface roughness in response to wind erosion, the relative roughness of surfaces as defined by the ratio of the change in topographic roughness to the median bed grain size has also been shown hereto be important. Ratios in sand beds are 1.71 times greater for experiments using fibres compared to those using beads, suggesting that fibres contribute towards the development of roughness elements at the surface, a pattern not seen in soil beds. While not investigating windblown surfaces, a similar trend is observed by Ingraffia *et al*. [70], who note that when microplastic fibres were present in soils, surface roughness and complexity was higher after rainfall events. They observed that the fibres formed a ‘diffuse fluff’ on the surface forming an additional micro-roughness element.

### (b) The effect of microplastic size, shape and concentration on topographic surface development

Where microplastics are present in soil, their size, shape and concentration probably control any influence they may have on topographic surface development in response to wind erosion. Akin to previous findings, the similarity of both size and shape of microplastic beads to the mineral sediment particles in the sand beds limits their effect on surface roughness [22,71]. Conversely, fibres have an observable influence upon the surface characteristics in sand beds. This is probably due to their flexibility, which enables them to mould around mineral sediment particles causing their entanglement into the matrix with the formation of larger, aggregated particles and hence an associated increase in surface roughness [13,22]. The effects of fibres on surface roughness are less obvious in the soil beds tested here where there is no observable influence on the characteristics of the soil surfaces either prior to or after wind erosion. Within soil beds, fibres are potentially either lying flat against the soil, or if they are embedded vertically, then horizontal resolution of the laser scanner could miss them.

While the relative size effects between the median microplastic and median soil particle diameter have been shown to be important controls on microplastic entrainment by wind [62,63], the effect of relative plastic size on topographic development has not been quantified. In sand beds $\Delta_{TR}/D_{50p}$, the average relative roughness is 2.34 and 1.22 as compared to 3.07 and 0.99 for soil beds containing beads and fibres, respectively. This suggests that the scale of the roughness developed is larger than the beads but similar in scale to that of the fibres. This will probably have an effect on how easily plastics could be eroded from bed surfaces, whereby fibres are likely to be more exposed to the wind flow and hence more likely to be entrained as compared to beads, which will essentially be ‘lost’ within the bed, and this may explain their lower enrichment ratios.

In these experiments, microplastic concentration has been shown to have no effect on results and hence appears to be less important than microplastic shape in the development of surface topography. The effect of microplastic concentration on soil–microplastic interactions has rarely been discussed, and while the concentrations reported here are similar to those found in natural soils [19], it is possible that the concentrations were not high enough to significantly influence the development of the surface topography. However, despite using concentrations of between 0.05 and 2% as compared to our ranges of between 0.004 and 0.104%, de Souza Machado *et al*. [22] observed that low concentrations of microplastics seemed to cause stronger effects on soil bulk properties than higher concentrations. They did not explicitly quantity these findings but postulated that at lower concentrations microplastic particles would interact more with the natural matter, while at higher concentrations processes influenced by microplastic–microplastic interactions become more relevant.

### (c) The effect of microplastic size, shape and concentration on soil surface roughness and microplastic flux

SSR plays a role in modifying exchanges between the terrestrial and atmospheric systems [42] and hence has the potential to influence the re-entrainment of plastics, which are thought to be present in variable concentrations in most soils [72,73]. We tested the relationship between microplastic enrichment ratios and indicators of SSR. The enrichment ratio for beads is slightly lower for the soil than the sand but a similar order of magnitude (1–10). However, using all indices, the surface roughness is greater for the soil than for the sand in beds containing beads. This reinforces the idea that beads have minimal effect on SSR in sand probably due the similarities in size and shape between the microplastic beads and the mineral grains. Atmospheric enrichment of beads entrained from sand surfaces is therefore more likely to be due to the low density and loose packing of the plastic spheres rather than the surface roughness characteristics [37,62,74]. Regardless of the substrate type, the enrichment ratios are higher for fibres, as has been reported elsewhere [75,76]. Surface roughness for sand containing fibres is greater than for sand containing beads, particularly when described using spatial roughness (*a*). Surface roughness for soils containing fibres is very similar to soils containing beads, but the atmospheric enrichment is considerably greater. This suggests that the interaction of fibres and soil particles may affect the extent to which the former can be incorporated into the bed or released to the atmosphere. Due to their elongated shape, fibres, especially in soil beds, are more likely to become trapped at the surface than beads since the fibre length is greater than the mean pore size (Engdahl, 2018; [6]). Trapped microplastic fibres that are exposed at the surface are more likely to affect spatial SSR. However, where fibres are only partially buried or entangled, the subaerial component will be exposed to flutter and axial stresses [77] (Coene, 1982; [78]), which may in turn place stresses on the surrounding soil particles leading to vibration or dislodgement and the subsequent release of the fibre. Anderson & Turner [79] suggest that rather than accumulate microplastics, sand dunes may be transient reservoirs of microplastic, particularly where the plastic particles, such as the fibres used in our study, are larger than the interstitial pore spaces between the mineral grains.

## 5. Conclusion

Surface roughness is an important property for predicting wind erosion potential. At larger scales (greater than dm), the spatial patterning of roughness elements strongly influences wind erosion; however, at smaller scales (mm), it has been suggested that metrics based on surface roughness height, such as TR, are more important [30]. Our results indicate that models for predicting the wind entrainment of microplastics could usefully include metrics for the relative size of the microplastic particles and the mineral substrate (e.g. *D*_i_/*D*_50_) as well as the associated indicators of surface roughness height. Further research using a wider range of sediment and plastic types is needed to fully quantify how the development of small-scale SSR affects the flux of plastics from soils.

## Data Availability

The enrichment ratio data are already published in [38]. The raw DEM data used to derive the metrics reported in the paper are included in an online repository accessible via searching for the first author (https://datacat.liverpool.ac.uk/cgi/search/advanced).
